# From Risk Stratification to Prevention of Myocardial Infarction: Integrating Imaging and Biomarkers in the Perioperative Setting

**DOI:** 10.3390/biomedicines14051023

**Published:** 2026-04-30

**Authors:** Jeong Yoon Jang, Jae Seok Bae, Yun-Ho Cho, Yujin Shin, Han Ra Choi, Jeong Rang Park, Min Gyu Kang, Hye-Ree Kim, Yong-Lee Kim, Hyo Jin Lee, Kye-Hwan Kim, Jin-Yong Hwang, Sung-Eun Park, Jong-Hwa Ahn

**Affiliations:** 1 Division of Cardiology, Department of Internal Medicine, Gyeongsang National University Changwon Hospital, Gyeongsang National University School of Medicine, Changwon 51472, Republic of Koreahiha-ki@hanmail.net (Y.-H.C.); yugene91@gmail.com (Y.S.); kns3114@naver.com (H.R.C.); 2 Department of Internal Medicine, Gyeongsang National University Hospital, Gyeongsang National University School of Medicine, Jinju 52727, Republic of Korea; park-jr@nate.com (J.R.P.); hrmanse@naver.com (H.-R.K.); hyozhin@naver.com (H.J.L.);; 3 Department of Radiology, Gyeongsang National University Changwon Hospital, Gyeongsang National University School of Medicine, Changwon 51472, Republic of Korea; uneyes@hanmail.net

**Keywords:** perioperative myocardial infarction, noncardiac surgery, coronary computed tomography angiography, biomarkers, cardiac troponin, natriuretic peptides, risk stratification, prevention

## Abstract

Perioperative myocardial infarction (MI) and myocardial injury after noncardiac surgery (MINS) remain major causes of postoperative morbidity and mortality, yet optimal perioperative cardiovascular risk stratification remains challenging. This narrative review examines how cardiovascular imaging and circulating biomarkers may be integrated to improve perioperative risk assessment and to support more individualized preventive strategies in patients undergoing noncardiac surgery. We reviewed major clinical guidelines, landmark perioperative cohort studies, and key investigations addressing coronary computed tomography angiography, coronary calcium burden, natriuretic peptides, and cardiac troponin in the perioperative setting. Available evidence suggests that imaging and biomarkers provide complementary information, with imaging primarily reflecting structural coronary disease burden and biomarkers reflecting myocardial stress, biological vulnerability, and perioperative injury. Such multimodal assessment may refine risk estimation beyond conventional clinical indices alone, particularly in selected intermediate-risk patients or those with uncertain functional capacity. However, important limitations remain. Current evidence is heterogeneous across study populations, testing strategies, and endpoints, and standardized pathways for integrating imaging and biomarkers into routine clinical decision-making are not yet established. In addition, cost-effectiveness, accessibility, and the extent to which improved risk discrimination translates into better perioperative outcomes remain uncertain. Overall, the integration of imaging and biomarkers offers a clinically relevant framework for moving from perioperative risk stratification toward prevention, but its practical implementation and outcome benefit require further prospective validation.

## 1. Introduction

Perioperative myocardial infarction (MI) and related cardiovascular events remain major causes of morbidity and mortality in patients undergoing noncardiac surgery. Accurate preoperative cardiovascular risk stratification is therefore essential for identifying vulnerable patients and guiding perioperative management. However, conventional approaches based on clinical risk indices and functional assessment alone often have limited discriminatory performance in real-world practice, particularly in patients with heterogeneous cardiovascular risk profiles.

Recent studies have highlighted the incremental value of advanced testing strategies beyond traditional clinical assessment. In a large prospective cohort, preoperative NT-proBNP provided significant prognostic information for perioperative cardiovascular events in patients undergoing noncardiac surgery, remaining independently associated with vascular death and myocardial injury after noncardiac surgery in multivariable analysis [1]. Accumulating evidence also suggests that coronary computed tomography angiography (CTA) may improve perioperative risk stratification by directly characterizing structural coronary disease burden and refining risk assessment beyond conventional functional testing alone [2,3,4]. Across prospective studies, this value has been observed in different but related clinical settings, including intermediate-risk noncardiac surgery, comparison with dobutamine stress echocardiography, and further risk refinement after treadmill testing [2,3,4]. At the same time, these studies were conducted in selected populations and should be interpreted in the context of differences in study design, comparator tests, and endpoint definitions. This issue is particularly relevant because perioperative myocardial ischemic events are frequently silent or present atypically owing to anesthesia, analgesia, and postoperative clinical complexity. Accordingly, many clinically important events may be missed unless systematic surveillance and risk-based evaluation are applied. The concept of myocardial injury after noncardiac surgery (MINS) has further expanded the clinical spectrum of perioperative ischemic injury by recognizing prognostically relevant postoperative troponin elevation even in the absence of overt ischemic symptoms [5,6]. In parallel, contemporary international guidelines have increasingly emphasized the role of biomarkers, selective noninvasive testing, and individualized perioperative cardiovascular evaluation [7,8].

Despite these advances, an important gap remains between improved risk prediction and effective prevention. Perioperative ischemic complications arise through diverse mechanisms, including obstructive coronary artery disease, plaque instability, oxygen supply–demand mismatch, tachyarrhythmia, hypotension, anemia, and perioperative inflammatory stress [6,9]. Although both imaging and biomarkers have demonstrated prognostic relevance, several important uncertainties remain unresolved, including the optimal strategy for integrating these modalities in routine perioperative care, the most appropriate target population for multimodal testing, the timing of assessment before surgery, and the cost-effectiveness and practical feasibility of broader implementation. In addition, prior studies have been performed in selected populations and differ in surgical risk profile, endpoint definitions, and testing protocols, which may limit generalizability across diverse clinical settings. Thus, improved risk discrimination should not be assumed to translate automatically into improved perioperative outcomes unless test results meaningfully alter clinical decision-making and management.

These unresolved issues define the central knowledge gap addressed in this review. Therefore, available evidence from selected cohort studies and guideline-supported biomarker strategies suggests that a more integrated approach combining anatomical imaging, functional assessment, and circulating biomarkers may provide a more comprehensive estimate of perioperative cardiovascular risk than conventional approaches alone. At the same time, such integration requires critical evaluation of its limitations, implementation barriers, and real-world applicability.

In this review, we summarize the contemporary evidence regarding perioperative MI and MINS, with particular emphasis on the complementary roles of cardiovascular imaging and circulating biomarkers in perioperative risk assessment. Rather than merely listing supportive findings, we also examine the limitations, inconsistencies, and practical challenges of current evidence, including uncertainty regarding standardized integration pathways and the limited prospective data demonstrating that testing-guided strategies improve clinical outcomes. We further discuss how these tools may help bridge the gap between risk stratification and prevention of perioperative myocardial infarction in patients undergoing noncardiac surgery.

## 2. Methods of Literature Review

This manuscript was conducted as a narrative review. A literature search was performed using PubMed and Google Scholar to identify relevant studies published in English on perioperative myocardial infarction, myocardial injury after noncardiac surgery, coronary computed tomography angiography, coronary calcium, natriuretic peptides, cardiac troponin, and perioperative cardiovascular risk stratification. Major international guidelines, landmark cohort studies, key prospective studies, and clinically relevant review articles were prioritized. Additional references were identified through manual review of reference lists from selected articles. The aim of this review was not to perform a formal systematic review or meta-analysis, but rather to provide a clinically focused and critical synthesis of the current evidence regarding integration of imaging and biomarkers in perioperative cardiovascular care.

## 3. Definitions and Clinical Spectrum of Perioperative Myocardial Infarction

Perioperative MI represents a clinically important ischemic complication occurring in the setting of noncardiac surgery and is associated with substantial short- and long-term adverse outcomes. In contrast to spontaneous MI in nonoperative settings, perioperative ischemic events frequently occur without typical chest pain or other classic ischemic symptoms because of anesthesia, sedation, analgesia, and the competing physiological stressors of the postoperative period [5,6]. As a result, reliance on symptoms alone may substantially underestimate the true burden of perioperative myocardial injury.

This recognition has led to broader attention to MINS, which refers to prognostically relevant postoperative troponin elevation attributed to ischemia, with or without accompanying symptoms or electrocardiographic changes [5,10]. The concept of MINS is particularly important because many patients with postoperative troponin elevation do not fulfill the full diagnostic criteria for spontaneous MI, yet still experience significantly higher short-term mortality [5,10]. The Fourth Universal Definition of Myocardial Infarction provides a useful framework for distinguishing myocardial injury from myocardial infarction by emphasizing that myocardial injury is defined by cardiac troponin values above the 99th percentile upper reference limit, whereas MI additionally requires evidence of acute myocardial ischemia [6]. Accordingly, perioperative myocardial injury is a broader biochemical concept, whereas perioperative MI refers to the subset of patients in whom myocardial injury is accompanied by clinical, electrocardiographic, imaging, or other evidence of acute ischemia. MINS occupies an important intermediate position in this framework, referring specifically to perioperative myocardial injury attributed to ischemia, even when the full diagnostic criteria for myocardial infarction are not met.

For clarity, we use the term “perioperative myocardial injury” to denote postoperative troponin elevation in general, “MINS” to denote ischemia-related perioperative myocardial injury of prognostic significance, and “perioperative MI” to denote myocardial injury that fulfills the diagnostic criteria for myocardial infarction. In perioperative practice, this distinction is essential because postoperative troponin elevation may reflect type 1 MI, type 2 MI caused by oxygen supply–demand mismatch, or nonischemic myocardial injury, and careful clinical interpretation is therefore required.

Accordingly, the clinical spectrum of perioperative myocardial ischemic events extends beyond overt infarction to include asymptomatic myocardial injury, silent ischemic troponin elevation, and clinically recognized perioperative MI. This broader spectrum better reflects the biology and prognostic relevance of postoperative ischemic injury and provides the conceptual basis for modern perioperative surveillance strategies [5,10].

## 4. Pathophysiology and Mechanisms of Perioperative Ischemic Events

Perioperative ischemic events arise through heterogeneous and often overlapping mechanisms. In some patients, perioperative MI is related to plaque rupture, plaque erosion, or coronary thrombosis superimposed on preexisting atherosclerotic disease, a mechanism conceptually similar to type 1 MI in nonoperative settings [6,10]. However, many perioperative events are thought to result from type 2 ischemic injury, in which myocardial oxygen demand exceeds oxygen supply during periods of physiological stress [6,10]. Surgery induces a unique milieu characterized by sympathetic activation, inflammatory responses, hemodynamic fluctuations, hypercoagulability, pain, anemia, hypoxemia, and fluid shifts, all of which may precipitate ischemia in susceptible patients.

This pathophysiological complexity has important clinical implications. Patients with anatomically significant coronary artery disease may be particularly vulnerable to perioperative hypotension, tachycardia, or anemia, whereas others may have relatively nonobstructive coronary anatomy but still develop myocardial injury in the presence of severe physiological stress or microvascular dysfunction [6,8,10]. Moreover, perioperative myocardial injury may coexist with arrhythmia, heart failure, or systemic complications such as sepsis and renal dysfunction, further complicating diagnostic interpretation.

Importantly, these mechanisms should not be viewed as isolated or static processes. Rather, perioperative ischemic injury often reflects the interaction between a pre-existing structural substrate of risk and superimposed perioperative physiological stressors. Coronary atherosclerosis may determine baseline vulnerability, while intraoperative hypotension, tachycardia, bleeding, or hypoxemia may acutely reduce oxygen supply or increase myocardial demand. In the postoperative period, persistent sympathetic activation, inflammatory response, hypercoagulability, anemia, and arrhythmia may further amplify this imbalance and contribute to ongoing myocardial injury.

Thus, the timing of injury is also clinically relevant. Preoperative disease burden may establish the background risk, intraoperative perturbations may trigger ischemic imbalance, and postoperative factors may sustain or worsen myocardial injury even in the absence of overt symptoms. This temporal and mechanistic overlap helps explain why perioperative myocardial injury is frequently multifactorial and why no single test fully captures risk across the perioperative continuum.

Taken together, these mechanisms suggest that perioperative risk cannot be fully understood through a single-domain approach. Anatomical coronary disease burden, myocardial reserve, neurohormonal activation, and perioperative physiological stress all contribute to event occurrence. This mechanistic heterogeneity helps explain why purely clinical models often have limited predictive precision and supports the need for more integrated approaches using both imaging and biomarkers [1,2,3,4,10].

## 5. Conventional Preoperative Cardiovascular Risk Assessment: Strengths and Limitations

Conventional preoperative cardiovascular assessment has traditionally relied on clinical history, comorbidity burden, estimated surgical risk, and functional capacity. The Revised Cardiac Risk Index (RCRI) remains one of the most widely recognized tools and incorporates six variables: high-risk surgery, ischemic heart disease, heart failure, cerebrovascular disease, insulin-treated diabetes, and elevated serum creatinine [11]. This simplified framework has had major practical value because it offers a rapid and reproducible estimate of perioperative cardiac risk. Contemporary guidelines also continue to emphasize a stepwise approach integrating urgency of surgery, active cardiac conditions, surgical risk, functional capacity, and selective testing rather than indiscriminate screening [7,8].

Despite these strengths, conventional assessment has important limitations. Clinical indices primarily reflect overt comorbidity and procedural risk but do not directly quantify coronary atherosclerotic burden, plaque characteristics, or subclinical myocardial vulnerability [7,8,11]. Functional capacity assessment may be imprecise, especially in elderly or frail patients, and symptoms are often confounded by noncardiac limitations. In addition, clinical scores do not fully account for the dynamic interaction between baseline cardiovascular disease and perioperative physiological stress. As a result, two patients with similar RCRI scores may differ substantially in coronary anatomy, hemodynamic reserve, and postoperative event risk.

For these reasons, conventional risk assessment should be viewed as the foundation rather than the endpoint of perioperative cardiovascular evaluation. It identifies broad risk categories, but it may not provide sufficient granularity to guide individualized prevention in intermediate-risk or diagnostically ambiguous patients [7,8,11,12].

## 6. Imaging-Based Risk Stratification in the Perioperative Setting

Noninvasive imaging has long played a role in perioperative cardiovascular evaluation, particularly when symptoms, functional limitation, or clinical risk factors raise concern for occult ischemic heart disease. Functional tests such as exercise treadmill testing, stress echocardiography, and myocardial perfusion imaging have traditionally been used to detect inducible ischemia and estimate perioperative risk [7,8]. These techniques can be clinically useful in selected patients, but each has practical and interpretive limitations related to exercise tolerance, image quality, operator dependence, and incomplete characterization of coronary anatomy.

Coronary computed tomography angiography (CCTA) offers a different and potentially complementary paradigm by directly visualizing coronary anatomy and total atherosclerotic burden. Rather than assessing ischemia alone, CCTA can identify obstructive stenosis, multivessel disease, and calcific atherosclerosis, thereby providing a more structural view of perioperative coronary risk [2,3,4,13]. However, the available evidence should be interpreted with caution, as prior studies have generally been conducted in selected surgical populations and differ in sample size, comparator modality, endpoint definition, and clinical context. In an earlier study, CCTA-based risk stratification was shown to predict perioperative cardiac events in patients undergoing intermediate-risk noncardiac surgery, supporting the concept that direct anatomical evaluation of coronary artery disease may improve preoperative cardiovascular risk assessment beyond conventional approaches [3]. In the PANDA trial, CCTA demonstrated superior prognostic accuracy compared with dobutamine stress echocardiography in patients undergoing noncardiac surgery, suggesting that direct anatomical assessment may be more informative than functional imaging in selected preoperative populations [2]. More recently, coronary CTA performed after treadmill testing was shown to provide incremental prognostic value, particularly in patients with positive exercise test results, reinforcing the role of anatomical refinement after initial functional screening [4]. At the same time, these studies do not establish that CCTA is uniformly superior in all perioperative settings, and their generalizability may be limited by local expertise, patient selection, and differences in perioperative care pathways.

In addition, CCTA has several practical limitations that should be considered in perioperative decision-making. These include radiation exposure, the use of iodinated contrast with potential risk in patients with impaired renal function, and the possibility of incidental extracardiac or coronary findings that may complicate interpretation or trigger additional testing. Furthermore, anatomical abnormalities identified by CCTA do not always translate directly into lesion-specific ischemia or management benefit, and therefore the clinical value of CCTA depends on appropriate patient selection and whether the findings are expected to alter perioperative care.

Additional evidence supports the prognostic importance of coronary calcium burden. In the ENCORES study, coronary calcium detected on existing nongated chest CT improved preoperative risk stratification before noncardiac surgery, highlighting the practical value of opportunistic calcium assessment even outside dedicated cardiac CT protocols [13]. Nevertheless, coronary calcium burden is an indirect marker of atherosclerotic risk and does not by itself define lesion-specific ischemia or guarantee clinical benefit from additional testing or intervention. Taken together, these studies support the potential role of imaging as a risk-refinement tool in selected perioperative patients, particularly when conventional clinical or functional assessment is inconclusive. However, the strength of evidence remains heterogeneous, and imaging should not be interpreted as uniformly superior or universally required across all perioperative settings. The major tools currently used for perioperative cardiovascular risk stratification, along with their respective strengths and limitations, are summarized in Table 1.

## 7. Biomarker-Based Risk Stratification

Circulating biomarkers provide a biologically complementary approach to perioperative risk assessment by capturing myocardial stress, subclinical injury, and neurohormonal activation. Among available markers, natriuretic peptides and cardiac troponins have the strongest evidence base in noncardiac surgery [1,5,9,14,15]. Preoperative BNP or NT-proBNP reflects underlying myocardial strain and hemodynamic vulnerability, whereas postoperative troponin surveillance helps identify ischemic injury that is often clinically silent but prognostically important.

The perioperative utility of natriuretic peptides has been supported by both meta-analytic and guideline-level evidence. An individual patient data meta-analysis demonstrated that preoperative and postoperative natriuretic peptide measurements improve prediction of death or nonfatal MI after noncardiac surgery [15]. Consistent with this, the Canadian Cardiovascular Society guideline recommended measuring BNP or NT-proBNP before surgery in selected higher-risk patients to refine risk estimation and guide postoperative troponin monitoring [14]. More recently, in a multicenter prospective cohort, NT-proBNP provided significant incremental prognostic value beyond treadmill testing for perioperative cardiovascular events, supporting its role as a clinically meaningful biomarker for preoperative risk refinement [1]. However, interpretation of natriuretic peptide levels requires clinical context, as elevations may reflect age, renal dysfunction, heart failure, or other systemic conditions rather than perioperative ischemic risk alone. In addition, study populations, assay thresholds, and perioperative endpoints vary across studies, which may limit standardization and broad applicability.

Cardiac troponin plays a somewhat different role. Rather than primarily functioning as a baseline screening tool, postoperative troponin measurement is central to detection of MINS and silent perioperative MI [5,9,10]. Large prospective data have shown that even modest postoperative elevations in high-sensitivity troponin are strongly associated with 30-day mortality, including in the absence of overt ischemic symptoms [9]. Nonetheless, troponin elevation is not synonymous with type 1 perioperative MI, and interpretation may be complicated by supply–demand mismatch, renal dysfunction, sepsis, heart failure, or other nonischemic causes of myocardial injury. Thus, biomarker-based strategies extend perioperative risk assessment beyond preoperative prediction alone and enable systematic postoperative event detection in patients who would otherwise go unrecognized.

## 8. Complementary Roles of Imaging and Biomarkers

A major conceptual advance in perioperative cardiovascular evaluation is the recognition that imaging and biomarkers measure different but highly complementary dimensions of risk. Imaging, particularly CCTA and coronary calcium assessment, characterizes the structural substrate of risk, including coronary stenosis, plaque burden, and extent of atherosclerosis [2,3,4,13]. Biomarkers, by contrast, reflect biological vulnerability, including myocardial wall stress, latent ventricular dysfunction, and ongoing or recent myocardial injury [1,5,9,14,15]. Neither domain alone fully captures the complexity of perioperative ischemic risk.

This complementarity is especially relevant in patients with intermediate clinical risk. A patient may have only modest symptoms and an equivocal treadmill test, yet CCTA may reveal extensive coronary disease or high calcium burden, suggesting substantial structural risk [2,3,4,13]. Conversely, a patient with nonobstructive coronary anatomy may still have elevated NT-proBNP, implying impaired cardiac reserve or subclinical myocardial dysfunction that increases perioperative vulnerability [1,15]. When both structural disease burden and biomarker-defined vulnerability are present, the overall probability of perioperative cardiac events is likely to be substantially higher than would be inferred from clinical assessment alone.

In this context, several prospective studies have illustrated a coherent multimodal framework across more than a decade: early work demonstrated the prognostic relevance of CCTA-based perioperative risk stratification in intermediate-risk noncardiac surgery [3], subsequent prospective comparison suggested that CCTA may offer additional prognostic information compared with dobutamine stress echocardiography in selected patients [2], more recent multicenter data confirmed the incremental prognostic value of NT-proBNP beyond treadmill testing [1], and contemporary evidence showed that CCTA may provide further prognostic refinement after treadmill testing [4]. Together, these findings support a model in which perioperative risk stratification is strengthened when anatomical imaging and biomarkers are used in a targeted, integrated manner rather than as isolated tests.

## 9. From Risk Stratification to Prevention

The clinical value of improved perioperative risk stratification lies not merely in prediction but in prevention. Identification of high-risk patients should prompt actionable measures such as optimization of guideline-directed medical therapy, careful perioperative hemodynamic management, individualized decisions regarding timing and intensity of monitoring, and structured postoperative surveillance [7,8,10,14]. Without a management response, even highly accurate risk prediction offers limited practical benefit.

Preventive strategies may include intensified control of heart rate and blood pressure, avoidance of prolonged hypotension and severe anemia, perioperative continuation or optimization of statin therapy when appropriate, careful management of antithrombotic therapy, and postoperative troponin surveillance in patients judged to be at increased risk [7,8,10,14]. In selected patients, preoperative identification of high anatomical coronary risk or markedly elevated natriuretic peptides may also justify closer postoperative telemetry, step-down or intensive monitoring, or early cardiology involvement. Importantly, such strategies should be individualized, because indiscriminate escalation of testing or therapy may increase cost and complexity without improving outcomes.

Therefore, the real promise of multimodal perioperative evaluation is that it can shift care from broad, population-level risk estimation to patient-specific prevention. The goal is not simply to label patients as high risk, but to use that information to guide actionable perioperative management. However, whether such risk-guided strategies consistently reduce perioperative MI, MINS, or mortality remains to be established in prospective interventional studies.

## 10. Proposed Integrated Clinical Approach

**Figure 1:**
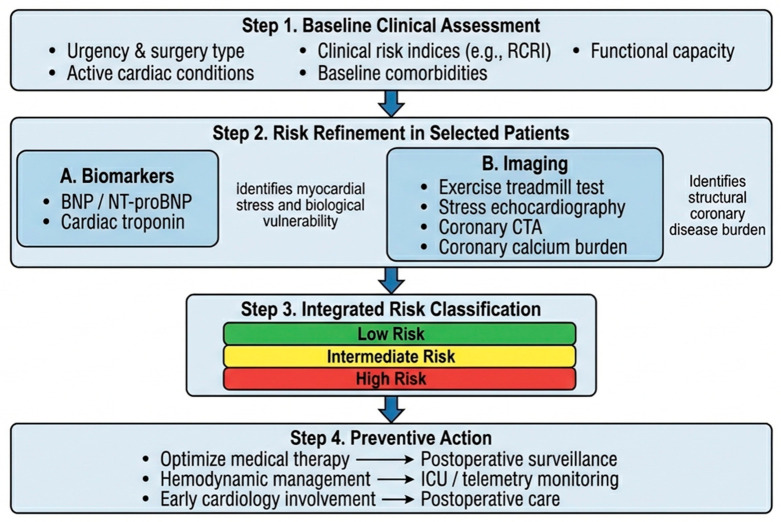
Proposed integrated framework for perioperative myocardial infarction risk stratification and prevention in patients undergoing noncardiac surgery. Perioperative cardiovascular evaluation begins with conventional clinical assessment, including surgical risk, active cardiac conditions, comorbidities, and functional capacity. In selected patients, biomarkers such as natriuretic peptides help assess myocardial vulnerability, whereas imaging modalities, particularly coronary computed tomography angiography, define the structural burden of coronary artery disease. Integrating clinical, biomarker, and imaging data may improve identification of high-risk patients and support individualized preventive strategies, including optimization of medical therapy, hemodynamic stabilization, postoperative troponin surveillance, and intensified perioperative monitoring, while emphasizing that multimodal testing should be selective and should not delay urgent surgery.

An integrated clinical approach to perioperative MI prevention can be conceptualized as a stepwise refinement process (Figure 1). First, all patients should undergo conventional clinical assessment using surgical urgency, active cardiac conditions, procedural risk, comorbidity profile, and functional status as the baseline framework [7,8,11]. This initial step helps distinguish patients who can proceed with routine perioperative care from those in whom further evaluation may be warranted, as well as those in whom additional testing is unlikely to alter management. Second, in patients with elevated clinical risk, uncertain functional capacity, or borderline findings on initial evaluation, selective natriuretic peptide testing may help identify individuals with heightened myocardial vulnerability [1,14,15]. Biomarker testing is particularly useful when additional biological risk information may influence postoperative surveillance intensity or perioperative monitoring. Third, when diagnostic uncertainty remains or when anatomical clarification is likely to influence perioperative management, CCTA may provide valuable information regarding coronary stenosis, plaque burden, and calcium burden [2,3,4,13]. However, advanced testing should be applied selectively, should not delay urgent surgery, and should generally be reserved for patients in whom the results are expected to affect clinical decision-making. The practical feasibility of this approach may vary according to institutional resources, local expertise, test availability, and the urgency of the surgical setting.

This framework is particularly relevant for patients with intermediate clinical risk, in whom management decisions are often most uncertain. In practice, such patients may include those with poor or uncertain functional capacity, positive or equivocal functional testing, elevated natriuretic peptides, or suspected substantial coronary risk. Low-risk patients may proceed with standard care, whereas very high-risk patients with unstable or active cardiac conditions may require treatment or postponement regardless of additional testing [7,8]. Similarly, advanced multimodal testing is unlikely to be useful when the results are not expected to change perioperative management. The greatest opportunity for multimodal refinement lies in the large intermediate-risk group, in whom biomarkers and imaging may reveal vulnerability not captured by conventional scoring systems alone. Postoperatively, patients with elevated preoperative biomarkers or significant imaging abnormalities may benefit from systematic troponin surveillance and closer monitoring to enable earlier detection and treatment of MINS or overt perioperative MI [5,9,10,14].

Although prospective intervention trials remain needed, a practical integrated pathway that combines clinical assessment, selected biomarkers, and anatomical imaging may better align perioperative cardiovascular evaluation with the actual mechanisms of perioperative ischemic injury. The value of such integration lies not only in improving risk discrimination but also in guiding decisions regarding postoperative surveillance, hemodynamic management, perioperative medical optimization, and early cardiology involvement in selected patients. At the same time, this strategy should not be interpreted as a routine or universally applicable pathway, because cost-effectiveness, accessibility, and implementation in real-world clinical practice remain incompletely defined. Such an approach offers a plausible bridge between contemporary risk stratification and meaningful prevention.

## 11. Discussion

This review highlights the evolving paradigm of perioperative cardiovascular evaluation from conventional clinical risk estimation toward a more integrated and individualized strategy. Although traditional approaches based on clinical risk indices, surgical risk, and functional capacity remain essential, they provide only a partial view of perioperative cardiovascular vulnerability [7,8,11,12]. Recent observational and guideline-supported evidence indicates that anatomical imaging and circulating biomarkers provide complementary information that may improve identification of patients at increased risk for perioperative MI and MINS, although the consistency and clinical applicability of this evidence vary across populations and testing strategies [1,2,3,4,13,14,15].

A central challenge in this field is the mechanistic heterogeneity of perioperative ischemic events. Some events are related to obstructive coronary artery disease and plaque-related complications, whereas others arise primarily from oxygen supply–demand mismatch during periods of hemodynamic stress [6,10]. This complexity helps explain why no single test can fully capture perioperative risk. Imaging modalities such as CCTA characterize structural coronary disease burden, whereas natriuretic peptides and troponin reflect biological vulnerability, myocardial stress, and perioperative injury [1,2,3,4,9,13,14,15]. However, complementarity should not be interpreted as proof that routine multimodal testing is universally warranted. Rather, the available evidence supports selective integration in clinically appropriate patients, while broader implementation remains constrained by cost, accessibility, local expertise, and uncertainty regarding whether additional testing meaningfully changes management.

Several prospective studies have illustrated this integrated concept across different perioperative settings. Early work demonstrated that CCTA-based risk stratification could predict perioperative cardiac events in intermediate-risk noncardiac surgery [3]. Subsequent prospective comparison suggested that CCTA may provide additional prognostic information relative to dobutamine stress echocardiography in selected patients [2]. More recent multicenter data showed that NT-proBNP added prognostic information beyond treadmill testing [1] and that coronary CTA may further refine risk assessment after treadmill testing [4]. Taken together, these findings support the view that perioperative risk stratification may be strengthened when structural and biological domains are assessed together, although the available evidence remains heterogeneous and does not define a single universally applicable testing pathway.

A key unresolved issue is that improved risk discrimination does not necessarily translate into improved clinical outcomes. A more precise understanding of perioperative cardiovascular risk may support better selection of patients for closer postoperative surveillance, intensified hemodynamic management, optimization of medical therapy, and early cardiology involvement [7,8,10,14]. However, evidence remains limited as to whether imaging- or biomarker-guided strategies actually reduce perioperative MI, MINS, or mortality, rather than simply identifying patients at higher risk. This distinction is particularly important in patients whose management remains uncertain after conventional evaluation, because the practical value of testing depends on whether it leads to actionable and effective prevention.

Another important limitation in the current literature is the absence of a standardized integration pathway. Existing studies vary in patient selection, surgical populations, endpoint definitions, biomarker protocols, and imaging strategies, which limits direct comparability and generalizability [1,2,3,4,5,13,14,15]. In addition, routine use of advanced imaging or biomarker testing in all surgical candidates is unlikely to be cost-effective or necessary, and interpretation of perioperative troponin elevation remains challenging when ischemic and nonischemic mechanisms may coexist [6,9,10]. These limitations underscore the need to move beyond descriptive prognostic studies toward prospective clinical pathways that test whether selective multimodal assessment can improve perioperative decision-making and patient outcomes.

Despite these limitations, the available evidence supports a shift away from a one-dimensional model of perioperative evaluation toward a more nuanced and mechanism-informed approach that better reflects the biology of perioperative ischemic injury [1,2,3,4,9,10,13,14,15]. The most promising future direction is not simply broader testing, but more selective and evidence-based integration of clinical assessment, imaging, and biomarkers into practical perioperative care pathways. In this regard, integrated evaluation may represent an important step toward more precise and preventive cardiovascular care in patients undergoing noncardiac surgery, provided that future studies define its cost-effectiveness, feasibility, and outcome benefit more clearly.

## 12. Conclusions

Perioperative myocardial infarction and MINS remain important causes of adverse outcomes after noncardiac surgery and are frequently under-recognized because of their silent or atypical presentation. Conventional clinical risk assessment provides an essential framework for perioperative evaluation but may not adequately capture the full spectrum of structural coronary disease and biological vulnerability contributing to perioperative ischemic events.

Available evidence indicates that imaging modalities, particularly CCTA, and circulating biomarkers such as NT-proBNP and cardiac troponin can provide complementary and clinically meaningful information beyond traditional assessment. By integrating these modalities, clinicians may achieve more precise perioperative cardiovascular risk stratification and better identify patients who may benefit from individualized preventive strategies and closer surveillance.

Overall, the integration of imaging and biomarkers offers a clinically relevant bridge between perioperative risk stratification and prevention. A multimodal, mechanism-informed approach may improve the precision of perioperative cardiovascular care and ultimately help reduce avoidable ischemic complications in patients undergoing noncardiac surgery.

## 13. Future Directions

Future research should focus on translating improved risk prediction into actionable and outcome-improving clinical strategies. Prospective studies are needed to determine whether multimodal approaches combining clinical assessment, imaging, and biomarkers can guide interventions that reduce perioperative MI, MINS, and mortality. Such studies should evaluate not only discrimination metrics but also clinical utility, feasibility, and cost-effectiveness.

Another important direction is the development of integrated perioperative risk models that combine structural, functional, and biomarker data into a unified framework for selective clinical application. These models may allow more precise identification of patients with distinct perioperative risk phenotypes and help guide tailored surveillance and prevention strategies. Advances in artificial intelligence and clinical decision support may further facilitate individualized perioperative cardiovascular assessment by integrating complex multimodal data in real time.

Standardization is also needed in the measurement and interpretation of perioperative biomarkers, especially postoperative troponin surveillance, as well as in the selective use of CCTA and coronary calcium assessment in preoperative pathways. Finally, future studies should clarify which subgroups of patients derive the greatest benefit from multimodal assessment, particularly among those with intermediate clinical risk, equivocal functional testing results, or discordant findings across testing domains.

As perioperative medicine continues to evolve, the greatest opportunity may lie not simply in predicting risk more accurately, but in using that information to deliver targeted, practical, and preventive care. In this regard, integrated imaging- and biomarker-guided evaluation represents a promising next step in perioperative cardiovascular medicine.

## Figures and Tables

**Table 1:** Summary of major tools for perioperative cardiovascular risk stratification in patients undergoing noncardiac surgery.

Modality	Main Purpose	Strengths	Limitations	Potential Clinical Role
Clinical risk indices (e.g., RCRI)	Baseline risk estimation	Simple, widely available, guideline-based, easy to apply	Limited granularity, does not directly assess coronary anatomy or myocardial vulnerability	First-step screening and baseline perioperative risk classification
Functional capacity assessment	Estimate physiological reserve	Noninvasive, clinically intuitive, commonly used in routine practice	Subjective, often inaccurate in elderly or frail patients, influenced by noncardiac limitations	Initial evaluation and selection of patients for further testing
Exercise treadmill testing	Detect exercise-induced ischemia	Widely available, low cost, provides functional information	Limited sensitivity/specificity, requires exercise ability, no direct anatomical information	Selected patients with suspected ischemia and preserved exercise capacity
Dobutamine stress echocardiography	Detect inducible ischemia and wall-motion abnormalities	Functional ischemia assessment, no radiation	Operator-dependent, limited by image quality, indirect assessment of coronary anatomy	Alternative functional testing in selected higher-risk patients
Coronary computed tomography angiography	Define coronary anatomy and atherosclerotic burden	Direct visualization of stenosis, plaque burden, multivessel disease, high negative predictive value	Radiation/contrast exposure, incidental findings, not necessary for all patients	Anatomical risk refinement, especially in intermediate-risk or diagnostically uncertain patients
Coronary artery calcium assessment	Estimate calcific atherosclerotic burden	Simple structural risk marker, may be derived from nongated CT, opportunistic use possible	Does not directly define lesion-specific ischemia, limited functional information	Additional structural risk stratification, especially when chest CT is already available
BNP/NT-proBNP	Identify myocardial stress and hemodynamic vulnerability	Strong prognostic value, useful for preoperative risk refinement, easy to measure	Nonspecific elevations in renal dysfunction, heart failure, age, and other systemic conditions	Preoperative biomarker-based risk refinement and selection of higher-risk patients for postoperative troponin surveillance and closer monitoring
Cardiac troponin	Detect myocardial injury	Sensitive marker of perioperative myocardial injury, central for MINS detection	Interpretation may be challenging without clinical context, not primarily an anatomical marker	Postoperative surveillance and early identification of silent perioperative myocardial injury, particularly in selected higher-risk patients

## Data Availability

No new data were created or analyzed in this study. Data sharing is not applicable to this article.

## References

[1] Duceppe E., Patel A., Chan M.T.V., Berwanger O., Ackland G., Kavsak P.A., Rodseth R., Biccard B., Chow C.K., Borges F.K. (2020). Preoperative N-Terminal Pro-B-Type Natriuretic Peptide and Cardiovascular Events After Noncardiac Surgery: A Cohort Study. Ann. Intern. Med..

[2] Ahn J.H., Jeong Y.H., Park Y., Kwak C.H., Jang J.Y., Hwang J.Y., Hwang S.J., Koh J.S., Kim K.H., Kang M.G. (2020). Head-to-head comparison of prognostic accuracy in patients undergoing noncardiac surgery of dobutamine stress echocardiography versus computed tomography coronary angiography (PANDA trial): A prospective observational study. J. Cardiovasc. Comput. Tomogr..

[3] Ahn J.H., Park J.R., Min J.H., Sohn J.T., Hwang S.J., Park Y., Koh J.S., Jeong Y.H., Kwak C.H., Hwang J.Y. (2013). Risk stratification using computed tomography coronary angiography in patients undergoing intermediate-risk noncardiac surgery. J. Am. Coll. Cardiol..

[4] Park J.R., Bae J.S., Lee J.M., Cho Y.H., Jang J.Y., Shin Y., Choi H.R., Kim Y.L., Yu G.I., Kwak C.H. (2026). Incremental prognostic value of coronary CTA after treadmill testing in noncardiac surgery candidates: Results from a multicenter prospective cohort. Prog. Cardiovasc. Dis..

[5] Botto F., Alonso-Coello P., Chan M.T., Villar J.C., Xavier D., Srinathan S., Guyatt G., Cruz P., Graham M., Wang C.Y. (2014). Myocardial injury after noncardiac surgery: A large, international, prospective cohort study establishing diagnostic criteria, characteristics, predictors, and 30-day outcomes. Anesthesiology.

[6] Thygesen K., Alpert J.S., Jaffe A.S., Chaitman B.R., Bax J.J., Morrow D.A., White H.D., Executive Group on behalf of the Joint European Society of Cardiology (ESC)/American College of Cardiology (ACC)/American Heart Association (AHA)/World Heart Federation (WHF) Task Force for the Universal Definition of Myocardial Infarction (2018). Fourth Universal Definition of Myocardial Infarction (2018). Circulation.

[7] Halvorsen S., Mehilli J., Cassese S., Hall T.S., Abdelhamid M., Barbato E., De Hert S., de Laval I., Geisler T., Hinterbuchner L. (2022). 2022 ESC Guidelines on cardiovascular assessment and management of patients undergoing non-cardiac surgery. Eur. Heart J..

[8] Thompson A., Fleischmann K.E., Smilowitz N.R., de Las Fuentes L., Mukherjee D., Aggarwal N.R., Ahmad F.S., Allen R.B., Altin S.E., Auerbach A. (2024). 2024 AHA/ACC/ACS/ASNC/HRS/SCA/SCCT/SCMR/SVM Guideline for Perioperative Cardiovascular Management for Noncardiac Surgery: A Report of the American College of Cardiology/American Heart Association Joint Committee on Clinical Practice Guidelines. Circulation.

[9] Devereaux P.J., Biccard B.M., Sigamani A., Xavier D., Chan M.T., Srinathan S.K., Walsh M., Abraham V., Pearse R., Wang C.Y. (2017). Association of postoperative high-sensitivity troponin levels with myocardial injury and 30-day mortality among patients undergoing noncardiac surgery. JAMA.

[10] Ruetzler K., Smilowitz N.R., Berger J.S., Devereaux P.J., Maron B.A., Newby L.K., de Jesus Perez V., Sessler D.I., Wijeysundera D.N., American Heart Association Council on Cardiopulmonary, Critical Care, Perioperative and Resuscitation (2021). Diagnosis and Management of Patients With Myocardial Injury After Noncardiac Surgery: A Scientific Statement From the American Heart Association. Circulation.

[11] Lee T.H., Marcantonio E.R., Mangione C.M., Thomas E.J., Polanczyk C.A., Cook E.F., Sugarbaker D.J., Donaldson M.C., Poss R., Ho K.K. (1999). Derivation and prospective validation of a simple index for prediction of cardiac risk of major noncardiac surgery. Circulation.

[12] Gupta P.K., Gupta H., Sundaram A., Kaushik M., Fang X., Miller W.J., Esterbrooks D.J., Hunter C.B., Pipinos I.I., Johanning J.M. (2011). Development and validation of a risk calculator for prediction of cardiac risk after surgery. Circulation.

[13] Choi D.Y., Hayes D., Maidman S.D., Dhaduk N., Jacobs J.E., Shmukler A., Aizer A., Min J.K., Ko J.P., Einstein A.J. (2023). Existing Nongated CT Coronary Calcium Predicts Operative Risk in Patients Undergoing Noncardiac Surgeries (ENCORES). Circulation.

[14] Duceppe E., Parlow J., MacDonald P., Lyons K., McMullen M., Srinathan S., Graham M., Tandon V., Styles K., Bessissow A. (2017). Canadian Cardiovascular Society Guidelines on Perioperative Cardiac Risk Assessment and Management for Patients Who Undergo Noncardiac Surgery. Can. J. Cardiol..

[15] Rodseth R.N., Biccard B.M., Le Manach Y., Sessler D.I., Lurati Buse G.A., Thabane L., Schutt R.C., Bolliger D., Cagini L., Cardinale D. (2014). The prognostic value of B-type natriuretic peptide in patients undergoing noncardiac surgery: Systematic review and individual patient meta-analysis. J. Am. Coll. Cardiol..

